# Prediction of optimal continuous positive airway pressure in Thai patients with obstructive sleep apnea

**DOI:** 10.1038/s41598-021-93554-5

**Published:** 2021-07-06

**Authors:** Narongkorn Saiphoklang, Kanyada Leelasittikul, Apiwat Pugongchai

**Affiliations:** 1 Division of Pulmonary and Critical Care Medicine, Department of Internal Medicine, Faculty of Medicine, Thammasat University, 99/209 Paholyotin Road, Pathum Thani, 12120 Thailand; 2 Medical Diagnostics Unit, Thammasat University Hospital, Pathum Thani, 12120 Thailand

**Keywords:** Medical research, Risk factors

## Abstract

Continuous positive airway pressure (CPAP) is simple and effective treatment for obstructive sleep apnea (OSA) patients. However, the CPAP prediction equation in each country is different. This study aimed to predict CPAP in Thai patients with OSA. A retrospective study was conducted in Thai patients, who OSA was confirmed by polysomnography and CPAP titration from January 2015 to December 2018. Demographics, body mass index (BMI), neck circumference (NC), Epworth sleepiness scale, apnea–hypopnea index (AHI), respiratory disturbance index (RDI), mean and lowest pulse oxygen saturation (SpO_2_), and optimal pressure were recorded. A total of 180 subjects were included: 72.8% men, age 48.7 ± 12.7 years, BMI 31.0 ± 6.3 kg/m^2^, NC 40.7 ± 4.1 cm, AHI 42.5 ± 33.0 per hour, RDI 47.1 ± 32.8 per hour, and lowest SpO_2_ 77.1 ± 11.0%. Multiple linear regression analysis identified NC, BMI, RDI, and lowest SpO_2_. A final CPAP predictive equation was: optimal CPAP (cmH_2_O) = 4.614 + (0.173 × NC) + (0.067 × BMI) + (0.030 × RDI) − (0.076 × lowest SpO_2_). This model accounted for 50.0% of the variance in the optimal pressure (R^2^ = 0.50). In conclusion, a CPAP prediction equation can be used to explain a moderate proportion of the titrated CPAP in Thai patients with OSA. However, the CPAP predictive equation in each country may be different due to differences of ethnicity and physiology.

Trial registration: TCTR20200108003.

## Introduction

Obstructive sleep apnea (OSA) is a potentially serious sleep disorder which is characterized by complete or partial obstructions of the upper airway during sleep resulting in repetitive episodes of reduction or elimination of airflow, pressure, and increasing respiratory muscle activities^1,2^. It is usually associated with sleep disruption, oxygen desaturation^2^, cognitive impairment^3^, increased daytime sleepiness^2^, and decreased concentration and quality of life^4,5^. There is increasing evidence to support that OSA can cause several diseases such as cardiovascular disease^6,7^, stroke^8,9^, and hypertension^10,11^. The estimated prevalence of OSA is about 4% in men and 2% in women^1^. There are many factors that significantly affect the OSA. Obesity is the strongest factor^12^. Moreover, sex (male), snoring, and personal physiology are also factors that affect OSA^13,14^.

Continuous positive airway pressure (CPAP) is recognized as the most effective, standard, and safe treatment for OSA patients^15^. It is extremely effective in eliminating apnea, hypopnea, respiratory effort related arousal, snoring, and for correcting oxygen level during sleep. Also, it helps decrease daytime sleepiness and enhance quality of life^16–18^.

Generally, CPAP pressure is performed in laboratory for finding the optimal pressure. This is called CPAP titration^19^. However, some patients cannot find the optimal pressure the first time and have to repeat the test. The procedure of finding the CPAP pressure is time consuming, labor intensive, expensive, and delays prescription. Predictive equation of optimal CPAP pressure is another method to find the pressure. It is a simple way of obtaining an optimal CPAP pressure in a short period of time^20^. The equation is derived from demographic, anthropometric, and polysomnographic variables to determine the pressure^21–23^.

However, the CPAP predictive equation is different in each country. Race or ethnicity and difference of physiology may play an important role in determining body size and severity of OSA. The objective of this study was to predict of the optimal CPAP pressure in Thai patients with obstructive sleep apnea.

## Methods

### Study design and subjects

We retrospectively reviewed the data of Thai patients with OSA who were at least 18 years old, male or female, and had successfully undergone polysomnography and CPAP titration at Thammasat University Hospital, Thailand from January 2015 to December 2018. All subjects had had OSA indicated by apnea–hypopnea index (AHI) ≥ 5 events per hour. They had been diagnosed in the first part of the night of the study and the optimal pressure had been determined in the second part. We excluded patients who had high residual AHI (AHI ≥ 5 events per hour) after was used CPAP. We included only 180 subjects for whom we had succeeded to find the optimal pressure. The optimal pressure was defined as reduction of AHI < 5 events per hour and had supine rapid eye movement (REM) for at least 15 min.

We recorded demographic variables: age and sex, anthropometric variables: height, weight, BMI, neck circumference (NC), polysomnographic variables: AHI, respiratory disturbance index (RDI), mean and lowest pulse oxygen saturation (SpO_2_), and optimal CPAP. Moreover, Epworth sleepiness scale (ESS) was also recorded.

Ethic approval was obtained from the Ethics Review Sub-Committee for Research Involving Human Research Subjects of Thammasat University, No.3 (IRB project No. 158/2561), in compliance with Declaration of Helsinki, The Belmont Report, CIOMS Guidelines and The International Practice (ICH-GCP). All methods were performed in accordance with these guidelines and regulations. All participants provided written informed consent.

### Polysomnography

Polysomnography (PSG) was performed according to established standard of American Academy of Sleep Medicine (AASM)^24,25^. It consisted of monitoring of sleep by electroencephalography (EEG), electrooculography (EOG), electromyography (EMG), electrocardiography (ECG), SpO_2_, body position, and snoring. In addition, airflow was monitored with oronasal thermistor and nasal transducer for detecting apnea and hypopnea. Respiratory effort was monitored with thoracoabdominal plethysmography.

### Respiratory events

Respiratory events were manually scored according to the 2012 AASM recommendations^25^. Apnea and hypopnea were defined by standard criteria^26^. Apnea was defined as decrement of airflow ≥ 90% from baseline for at least 10 s. Hypopnea was defined as reduction of nasal pressure ≥ 30% from baseline for at least 10 s and with ≥ 3% oxygen desaturation or accompanied by an EEG arousal for 3 to 15 s. Respiratory effort related arousal (RERA) was defined as a sequence of breath that reduces of nasal pressure < 30% from baseline for at least 10 s and is followed by an EEG arousal. AHI was defined as the total number of apnea and hypopnea per hour during sleep. RDI was calculated by the total number of apnea, hypopnea, and RERA per hour during sleep. Oxygen desaturation index (ODI) was defined as the number of events per hour in which oxygen saturation decreased by ≥ 3% from baseline^25^. The severity of OSA was determined by AHI or RDI; mild (AHI or RDI ≥ 5–14.9), moderate (AHI or RDI 15.0 – 29.9), and severe (AHI or RDI ≥ 30.0).

### CPAP titration

CPAP titration was performed by a trained polysomnographic technologist. The pressure was started at 4 cmH_2_O and the pressure was increased at least 1 cmH_2_O if there were 2 apneas, 3 hypopneas, 5 RERAs, or 3 min of loud snoring within 5 min. The pressure was increased until respiratory events were abolished. The optimal pressure was that which can control respiratory events while sleeping in supine position and included supine REM for at least 15 min (AHI < 5 events per hour).

### Statistical analysis

Data is presented as mean ± standard deviation and number (%). All statistical analyses were performed using SPSS version 23.0 software (IBM Corp., Armonk, NY, USA). Pearson’s correlation coefficient was used to explore the relationship between the optimal pressure and all variables. Multiple linear regression by backward stepwise method was used to identify independent predictive variables and to develop a predictive equation of the optimal CPAP. A two-sided *P* value < 0.05 was considered statistically significant.

## Results

**Figure 1:**
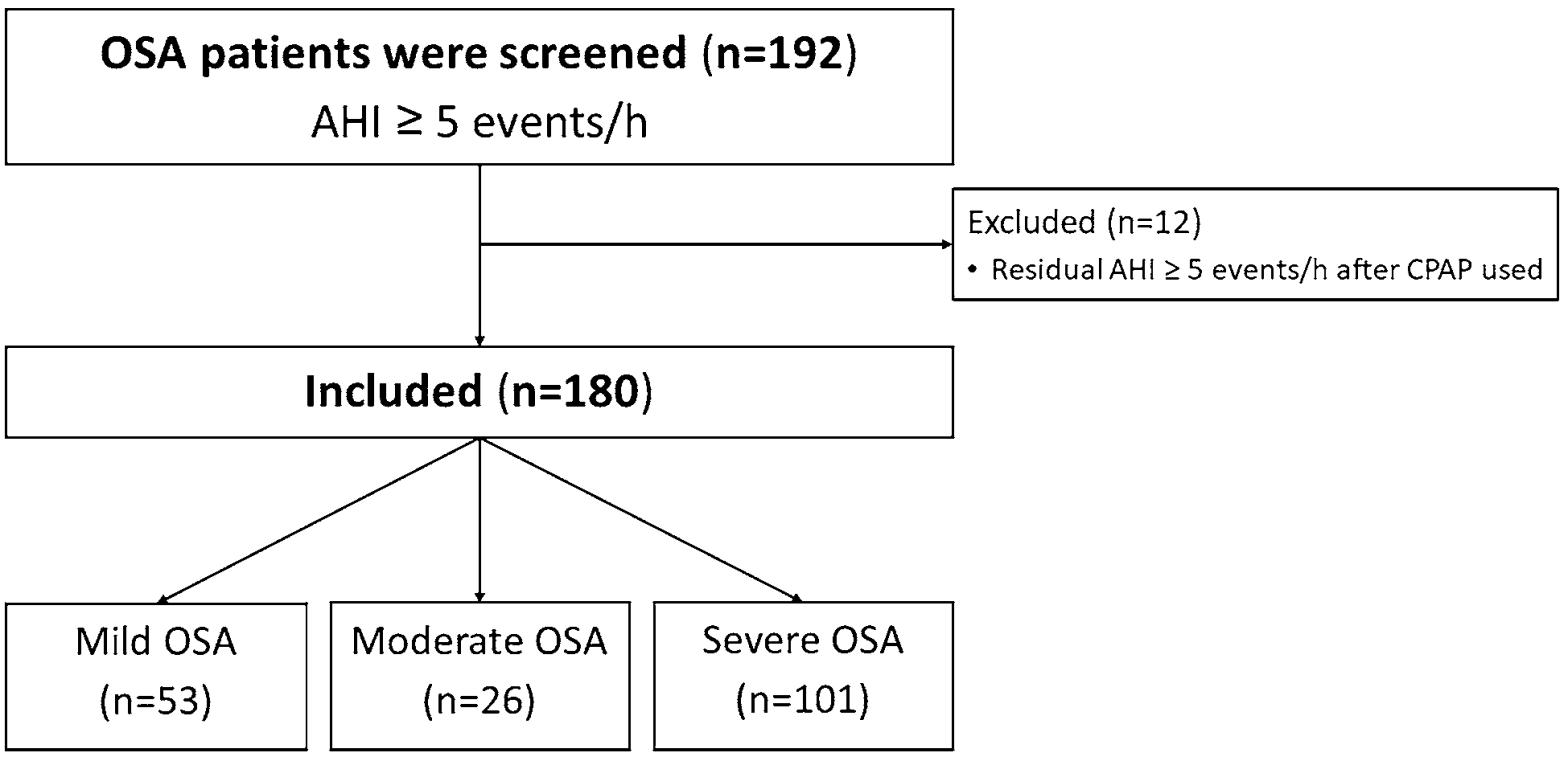
Flowchart of the study in OSA patients. *AHI* Apnea–hypopnea index, *CPAP* Continuous positive airway pressure, *OSA* Obstructive sleep apnea.

One hundred and ninety-two subjects were initially screened, with 12 subsequently excluded from analysis. A total of 180 subjects were included in the final analysis (Fig. 1). The demographic, anthropometric, and polysomnographic variables are shown in Table 1.

**Table 1:** Correlations between the optimal pressure and baseline characteristics, and polysomnographic parameters in 180 patients with obstructive sleep apnea.

Parameter	Data	Correlation coefficient (*r*)	*P* value
Male/female	131 (72.8)/49 (27.2)	NA	NA
Age, years	48.71 ± 12.34	− 0.073	0.328
Body mass index, kg/m^2^	31.04 ± 6.33	0.458	< 0.001
Neck circumference, cm	40.72 ± 4.06	0.513	< 0.001
Systolic blood pressure, mmHg	133.43 ± 16.63	0.131	0.079
Diastolic blood pressure, mmHg	83.54 ± 11.59	0.169	0.023
Heart rate, beats/minute	75.29 ± 13.56	0.200	0.007
Epworth sleepiness scale, score	10.64 ± 4.68	0.130	0.082
Apnea hypopnea index, events per hour	42.50 ± 32.98	0.586	< 0.001
Respiratory disturbance index, events per hour	47.08 ± 32.80	0.577	< 0.001
Mean SpO_2_ during sleep, %	90.17 ± 5.85	− 0.517	< 0.001
Lowest SpO_2_ during sleep, %	77.11 ± 10.69	− 0.470	< 0.001
Oxygen desaturation index, events per hour	11.50 ± 9.29	0.523	< 0.001
Optimal pressure, cmH_2_O	9.32 ± 3.01	NA	NA

Data shown as mean ± SD or n (%).

*NA* Not applicable, *SpO*_*2*_ Pulse oxygen saturation.

### The correlation between the optimal pressure and demographic, anthropometric, and polysomnographic variables

Table 1 shows significant correlation between the optimal pressure and standard parameters. The results found that the optimal pressure was positively correlated with BMI, NC, diastolic blood pressure, heart rate, AHI, RDI, and ODI. In addition, the optimal pressure was negatively correlated with mean SpO_2_, and lowest SpO_2_ during sleep.

### The CPAP predictive equation

The multiple linear regression by backward stepwise technique was used to identify independent predictive variables. A constant was 4.614. It indicated that NC (ß = 0.223, SE = 0.054, *P* = 0.003), BMI (ß = 0.150, SE = 0.034, *P* = 0.039), RDI (ß = 0.309, SE = 0.006, *P* < 0.001), and lowest SpO_2_ (ß = − 0.230, SE = 0.035, *P* = 0.001) were independent predictors of the optimal pressure. We used these parameters to develop a CPAP equation:

Optimal CPAP (cmH_2_O) = 4.614 + (0.173 × NC) + (0.067 × BMI) + (0.030 × RDI) − (0.076 × lowest SpO_2_).

This model accounted for 50% of the total variance (R^2^ = 0.50).

## Discussion

Our study is the first retrospective study of CPAP prediction in Thai OSA patients. Our study showed that obesity (BMI and NC) and severity of OSA (RDI and lowest SpO_2_) were independent factors affecting the predictive optimal pressure. In adults, the most common cause of OSA is obesity which is associated with soft tissue of the mouth and throat. During sleep, throat and tongue muscles are more relaxed especially in REM. This soft tissue can cause the airway to become blocked^27^. Moreover, obesity is related to the fat deposition in the body, especially deposits around the neck resulting in more NC. These affect the severity of the OSA. If OSA is more severe, there is increased chance of higher CPAP pressure.

This study was similar to several previous studies. Loredo et al.^21^ generated a CPAP prediction formula from 67 patients in USA. They found that BMI, lowest SpO_2_, and mean SpO_2_ affect the prediction of their formula. Their final CPAP equation was 30.8 + (0.03 × RDI) − (0.05 × lowest SpO_2_) − (0.2 × mean SpO_2_). It explained 67% of variance. Besides, the study of Miljeteig et al.^22^ examined the factors that account for the variability in CPAP level in 38 OSA patients in Canada. They revealed that NC, BMI, and AHI were independent factors for their CPAP prediction formula. Their final CPAP equation was − 5.12 + (0.13 × BMI) + (0.16 × NC) + (0.04 × AHI). It explained 76% of variance. Furthermore, Lin and colleagues^23^ determined CPAP prediction from a 121 member Taiwanese population. They found that BMI and AHI were significant predictors of effective CPAP. Our study and the three previous studies^21–23^ showed measure of obesity (NC and BMI) and severity of OSA (AHI, RDI, and lowest SpO_2_) affected the generated the CPAP prediction formula. Although, the variables were similar, the final CPAP model was different. This may be due to the differences of ethnicity and physiology of the patients in each country.

However, our study found that ESS and sex were not significant predictive factors for our CPAP prediction model. This was similar to the study of Stadling et al.^28^. In contrast, some previous studies showed that ESS and sex were significant for their CPAP prediction models. Lee et al.^29^ developed an equation for optimal CPAP based on data from 178 Korean patients. They revealed that the degree of daytime sleepiness assessed by ESS was one of the independent predictors of their CPAP prediction model. Their final model was 6.656 + (0.156 × BMI)-(0.071 × lowest SpO_2_) + (0.041 × RDI) + (0.094 × ESS). Also, a study of Yong and colleagues^30^ developed a prediction equation for optimal CPAP pressure from 92 Malaysian patients. They reported that ESS and sex (male) were independent variables for their CPAP model. Their final model was 13.666 + (2.361 × male) + (0.154 × ESS) − (0.059 × lowest SpO_2_). In addition, a study of Schiza et al.^31^ evaluated the effect of sex on a CPAP predicting equation and they found that sex was a statistically significant factor to predict optimal pressure.

Our formula provided an optimal CPAP estimation of pressure for a moderate proportion (50% of variance) of subjects. CPAP prediction formula can potentially be useful for improving CPAP titration, as a guide for setting home auto CPAP, or for final adjustment of attended CPAP titration. Similarly, Rowley JA et al. reported that use of a CPAP prediction model increased the percentage of successful of CPAP titration from 50 to 68%^32^.

Our study did have limitations. Firstly, this study is limited by its retrospective nature, thus some variables might not have been collected. Secondly, the small sample size may limit the power of the regression model in detecting other potential predictors of optimal pressure. Lastly, a final predictive CPAP equation was not tested prospectively in a PSG laboratory. Therefore, a prospective cohort study is needed to verify this equation in the future.

## Conclusions

A moderately predictive CPAP equation (50% of variance) can easily be derived from parameters of Thai patients with OSA. However, the CPAP predictive equation in each country may be different due to differences of ethnicity and physiology.
